# Multimodal Imaging to Identify Brain Markers of Human Prosocial Behavior

**DOI:** 10.1523/ENEURO.0304-24.2025

**Published:** 2025-02-28

**Authors:** Toru Ishihara, Hiroki Tanaka, Toko Kiyonari, Tetsuya Matsuda, Haruto Takagishi

**Affiliations:** ^1^Graduate School of Human Development and Environment, Kobe University, Kobe, Hyogo 657-8501, Japan; ^2^Brain Science Institute, Tamagawa University, Machida, Tokyo 194-8610, Japan; ^3^School of Social Informatics, Aoyama Gakuin University, Sagamihara, Kanagawa 252-5258, Japan

**Keywords:** economic game, MRI, myelin map, prosocial behavior

## Abstract

How humans achieve such a high degree of prosocial behavior is a subject of considerable interest. Exploration of the neural foundations of human prosociality has garnered significant attention in recent decades. Nevertheless, the neural mechanisms underlying human prosociality remain to be elucidated. To address this knowledge gap, we analyzed multimodal brain imaging data and data from 15 economic games. The results revealed several significant associations between brain characteristics and prosocial behavior, including stronger interhemispheric connectivity and larger corpus callosum volume. Greater functional segregation and integration, alongside fewer myelin maps combined with a thicker cortex, were linked to prosocial behavior, particularly within the social brain regions. The current study demonstrates that these metrics serve as brain markers of human prosocial behavior and provides novel insights into the structural and functional brain basis of human prosocial behavior.

## Significance Statement

The objective of this study was to identify brain markers associated with human prosocial behavior using data from 15 major economic games and multimodal magnetic resonance imaging data. The results of the study suggest that specific brain indicators, including myelin density and resting-state interhemispheric functional connectivities, are associated with prosocial behavior. The use of a data-driven approach holds particular significance within the realm of social neuroscience, a field that grapples with a multitude of variables.

## Introduction

Natural disasters cause extensive loss of life and damage to the lives of residents. Globally, people have visited affected areas to help survivors, clear debris, and raise funds for reconstruction. Such prosocial behavior toward genetically unrelated individuals distinguishes humans from other animals (Bowles and Gintis, 2011; Nowak, and Highfield, 2012; Tomasello et al., 2012). Human prosocial behaviors include altruism, such as volunteerism and charitable donations, fairness in resource distribution, cooperative behavior in collaboration, and reciprocity of favors. This leads to the following questions: why do humans exhibit more prosocial behavior than other species, and what are the critical factors distinguishing humans from other species? A possibility is that the function and structure of the human brain have evolved differently. Although the human brain is not larger than that of other animals, the percentage of its neocortex is known to be extremely high (Rilling and Insel, 1999), which is related to the degree of social networking (Dunbar and Shultz, 2007). In a social environment, which includes that of strangers, complex cognitive processing is undoubtedly necessary for building cooperative relationships. For example, the temporoparietal junction (TPJ) is the region involved in the cognitive ability to understand the mental states of other people, known as mentalizing, and higher-order mentalizing ability is prominent among humans (Saxe, 2006; Van Overwalle and Baetens, 2009). Other brain regions involved in understanding other people, such as empathy and the judgment of their emotions, include the temporal lobe, insular cortex, and inferior frontal gyrus (Blakemore, 2008). Many studies have revealed neural mechanisms behind human prosocial behavior; neural activation in the social brain regions forms the neural basis of human prosocial behavior (Morishima et al., 2012; Kameda et al., 2016). Despite the major progress made in understanding the neural mechanisms underlying human prosocial behavior, the full extent of the neural basis of prosocial behavior is yet to be fully elucidated. Since most studies have only examined relationships between single brain regions or relatively few regions utilizing limited neuroimaging methodologies, such as volumetric and activation techniques, further investigations are needed to fully understand the complex neural networks and processes that support human prosocial behavior. Considering the brain as a single system and elucidating its functioning for prosocial behavior are important for understanding the evolution of human prosocial behavior. Therefore, we aimed to use data-driven analysis of multimodal magnetic resonance imaging (MRI) data to identify brain functions and structures that underlie prosocial behavior.

## Materials and Methods

### Experimental model and study participant details

This study was a secondary analysis of data collected in an earlier research project (Neuropsychological and Social Institutional Foundations for Prosocial Behavior, http://www.human-sociality.net/english/). Approximately 400 men and women aged 20–60 years and living in Tokyo participated in repeated experiments from 2012 to 2018. They participated in 10 experiments, where they played various economic games, performed cognitive tasks, answered psychological questionnaires, underwent MRI, and provided saliva and buccal cell samples. Experiments were conducted multiple times to reduce the burden on participants and the overcarry effect in the economic game. The schedule for each experiment is shown in Table 1. To avoid arbitrary economic game selection, we conducted the analysis using behavioral data collected from all economic games. However, as the number of participants decreased after the 10th wave, the analysis was conducted only for the economic games conducted in the 2nd through 8th waves. The experimental protocol was approved by the Tamagawa University Ethics Committee (approval number, TRE18-030), and all participants completed a consent form before participating in each experiment. Among all participants, 217 who participated in all economic games and underwent MRI were included. The data collected in this project have been reported in previous literature (Extended Data Table 1-1). However, to the best of our knowledge, this study is the first to conduct a comprehensive analysis using data from all economic games and MRI scans.

**Table 1.** Schedule for collecting the data used in this study

Wave	Number of participants	Experiment period	Collected data
1	564	May 17, 2012–July 22, 2012	Demographic data
2	483	October 6, 2012–February 16, 2013	PDG-I
3	489	April 27, 2013–June 22, 2013	DG
			Faith game
4	474	September 2, 2013–October 26, 2013	PGG-I
			PDG-II
			SPPG
5	471	December 16, 2013–February 23, 2014	TG
			UG
6	470	May 10, 2014–July 27, 2014	TPPG-I
			TPPG-II
			Preemptive strike game
7	451	October 25, 2014–January 25, 2015	CG
8	424	September 15, 2015–December 12, 2015	PGG-II
			PGG with punishment
			Stag hunt game
9	290	November 21, 2016–March 3, 2018	MRI data (T1w, T2w, resting-state fMRI, diffusion MRI)
10	307	July 16, 2018–November 24, 2018	

The list of papers published in this research project is shown in Extended Data Table 1-1.

### Method details

#### Economic games

Every participant successfully participated in 15 economic games conducted under complete anonymity. The number of participants in each session ranged from 2 to 10, and each participant worked in an individual booth. All experiments did not use deception, and participants received rewards based on their actual behavior. Descriptions of the variables measured in each economic game and used in the analysis are presented in Extended Data Table 1-2. For each economic game, participants were provided with feedback on the decisions of other players at the end of the game. Rewards were determined based on the actual decisions of the participants.

1. Prisoner's dilemma game I
  Prisoner's dilemma game I (PDG-I) was conducted in pairs. Participants had to choose whether to give their opponent the endowment received from the experimenter. The opponent's endowment was doubled and then given to the other player. Participants underwent nine PD trials with a different partner each time. There were three experimental conditions: simultaneous PDG (where both participants made decisions at the same time), sequential PDG first (where participants decided first, followed by the opponent), and sequential PDG second (where the opponent decided first, followed by participants). These three situations were each repeated thrice in a random order. In sequential PDG second, participants answered what decision they would make if their partner offered or did not offer using the strategy method. The endowment received from the experimenter was JPY 300, JPY 800, or JPY 1,500, and it was provided to participants in random order.
2. Dictator game
  Dictator game (DG) was conducted in pairs, with one participant playing the role of a dictator and the other of a recipient. Participants initially performed one-shot DG. All participants were first assigned to the role of the dictator and were asked to decide how much from JPY 1,000 to allocate to their partner. After the decision, the participant's role and partner were randomly determined, and the reward amount was determined based on the participant's actual behavior. After the game, participants were instructed to perform repeated one-shot DG. Participants then played DG six times, changing opponents each time they acted as a dictator. The endowment given to the dictator could be JPY 300, JPY 400, JPY 600, JPY 700, JPY 1,200, or JPY 1,300, and it was offered in a random order.
3. Faith Game
  Faith game was played in pairs. As in DG, there were two roles: the distributor, who distributed a certain amount of money, and the recipient, who received the distributed money. The money distributed by the distributor was tripled and given to the recipient. Participants decided the amount of the endowed JPY 1,000 (from the experimenter) they were certain to receive and the amount that they were willing to leave to the distributor for distribution as a recipient. If participants thought that the distributor would distribute more money to them, they gave more money to the distributor. Conversely, if the participant believed that the distributor would not allocate additional funds to them, they opted to retain a larger portion of the initial endowment of JPY 1,000.
4. Public goods game I
  Public goods game I (PGG-I) was played with several participants divided into groups of four or more. They decided in increments of 100 whether they were willing to contribute JPY 1,000 received from the experimenter for the group. Money contributed to the group was tripled and distributed equally among all participants. The experiment was completed in a single trial.
5. Prisoner's dilemma game II
  Prisoner's dilemma game II (PDG-II) was played in pairs. Participants decided the amount of the endowed JPY 1,000 they would provide to their partner, in increments of JPY 100. The endowment offered was doubled and then given to the opponent. The opponent was also determined at the same time. The experiment was completed in a single trial.
6. Second-party punishment game
  Participants played a one-shot, two-person second–party punishment game (SPPG) that comprised two phases—PDG and punishment. In the PDG phase, participants were randomly matched with another participant and played PDG with the opponent, while aware that both players would have a chance to reduce the other player's earnings by spending some of their money, up to JPY 1,500. In the PDG phase of the SPPG (PD/SPPG), each player was endowed JPY 1,000 and instructed to decide the amount of the endowed money they were willing to give their opponents (in increments of JPY 100). The money given was doubled and transferred to the opponent. The task was symmetrical, such that the player would receive twice the amount of money their opponent gave as well. Therefore, each player received the portion of the endowment that they did not give, plus twice the amount of money their opponent gave. When participants played this game, they knew that they would both have a chance to subtract money from the other player's cumulative earnings.
  When PD/SPPG was over, each participant was informed of their opponent's choice and was then asked to decide the amount (up to JPY 1,500, in increments of 100) they would spend to reduce their opponent's earnings. The strategy method was used to determine the expenditure for punishment; in other words, each participant was provided with a list of possible opponent cooperation levels, ranging from JPY 0 to JPY 1,000, in increments of 100; each participant was asked to decide the amount to spend in each case. No extra money was provided specifically to use for this spending. The money came from their earnings in earlier tasks and the show-up fee, although the participants were not informed about their accumulated earnings amount at that time. The amount spent was doubled, and this new amount was then subtracted from the total earnings (including earnings from other tasks) of the opponent. Participants were explicitly told that the amount of money taken away from their opponents would not be transferred to them.
7. Trust game
  Trust game (TG) was played in pairs: a trustor and a trustee. The trustor was asked to decide the amount of the endowed JPY 1,000 to be transferred to the trustee in increments of 100. The amount of money transferred was tripled and received by the trustee. Next, the trustee was asked to divide the money between themselves and the other participant. The decisions of the trustee were analyzed using the strategy method, which determined how the trustee would return to every decision the trustor could make, in increments of 10%. All participants in the experiment first made decisions as a trustor, then changed partners, and made decisions as trustees. The game ended after the decision as a trustee.
8. Ultimatum Game
  Ultimatum game (UG) was conducted in pairs: a proposer and a responder. First, the proposer received JPY 1,500 from the experimenter and proposed a plan to divide it between themselves and the responder in increments of 100. After the proposer's decision was made, the responder decided whether to accept or reject the proposal. If the responder accepted the proposal, both participants received the money as proposed. In case of rejection of the proposal, neither received anything. In the experiment, all participants first took the role of the proposer, followed by the change of partners and decision as a responder. The responder's decision was made using the strategy method, i.e., acceptance or rejection of all proposals that the proposer could make (from JPY 0 to JPY 1,500). The experiment was over when the decision as a responder was completed.
9. Third-party punishment game I
  Third-party punishment game I (TPPG-I) was played by three players. First, the distributor distributed the JPY 1,500 received from the experimenter between himself and the recipient in 100 increments. The recipient received the amount decided by the distributor. After the distributor's decision, a third party decided whether to reduce the distributor's money. Three times the amount of money spent by the third party was deducted from the distributor's amount. The third party could spend between JPY 0 and JPY 500 using money earned in another experiment. In the experiment, participants first played the role of the third party and used the strategy method to decide the amount they wanted to spend to punish the distributor for all decisions that they could take. After the third-party decisions were made, the next trial began, and three new participants were matched. The participants acted as distributors and decided how to distribute the JPY 1,500 between themselves and the recipients, following which three new people were matched; then participants played the role of a recipient and received the money distributed by the distributor, at which point the experiment ended.
10. Third-party punishment game II
  Third-party punishment game II (TPPG-II) differed from TPPG-I as the source used by the third party to deduct the distributor's money was the amount received from the experimenter (JPY 500). All other procedures were identical.
11. Preemptive strike game
  Preemptive strike game was played in pairs (Player 1 and Player 2). The buttons were displayed on each other's personal computers (PCs). If no one pressed the button for 30 s, both players received JPY 1,500. However, if Player 2 pressed the button first, Player 1 received JPY 500, and Player 2 received JPY 1,300 and vice versa. If either one of the players pressed the button, it showed up on the screen indicating that the other player would not be able to press the button. In other words, only one of the players could press the button. The experiment involved four conditions in which both parties received different amounts of money, which were presented to the participants in a random order. The matching partner changed each time. Condition 55: As described above. Condition 1010: If Player 2 pressed the button first, Player 1 received JPY 1,000, and Player 2 received JPY 1,300. If Player 1 pressed the button first, Player 1 received JPY 1,300, and Player 2 received JPY 100. Condition 510: If Player 2 pressed the button first, Player 1 received JPY 500, and Player 2 received JPY 1,300. If Player 1 pressed the button first, Player 1 received JPY 1,300, and Player 2 received JPY 1,000. Condition 105: If Player 2 pressed the button first, Player 1 received JPY 1,000, and Player 2 received JPY 1,300. If Player 1 pressed the button first, Player 1 received JPY 1,300, and Player 2 received JPY 500.
12. Chicken game
  Chicken game (CG) was played in pairs. Participants could choose to “proceed” or “turn back” simultaneously. If both participants decided to “proceed,” they received JPY 0. If both participants chose to “turn back,” the amount of money both participants received was JPY 300. If one chose to “proceed” and the other chose to “turn back,” the one who chose to “proceed” received JPY 1,200, and the one who chose to “turn back” received JPY 300. The game was played thrice, each time with a different opponent, and three different amounts were received based on the decisions of both players (Extended Data Table 1-2). These three payoff matrices were presented to the participants in a random order. Next, the participants were paired with a new partner. They played a game in which the participant decided first, and the opponent decided later (participant-first condition). In this case, Payoff Matrix 1 was used as the reward combination.
13. Public goods game II and public goods game with punishment
  Participants played a one-shot public goods game II (PGG-II). The rules of the game were the same as those of PGG-I. Next, participants were told that they would be playing a one-shot PGG with punishment. In this game, the player who offered the lowest amount in PGG was punished. The amount for punishments was deducted from the funds for the punishment system, which was tallied by each player. Twice the sum of the amounts was deducted from the player with the lowest contribution. Each player could contribute between JPY 0 and JPY 300 in increments of 10 from the rewards earned in the experiment to fund the punishment system. The game ended when the amount of money for punishment was determined.
14. Stag hunt game
  Stag hunt game was conducted in pairs. Participants decided whether to invest or keep the JPY 500 they received from the experimenter. If both participants decided to invest, they received JPY 1,000. If both decided to keep it in hand, both received JPY 500. If one participant decided to invest and the other decided to keep the money, the one who decided to invest the money received JPY 0, while the participant who decided to keep the money received JPY 500. Both decisions were made simultaneously, and the game ended with a single decision.

### MRI data acquisition, preprocessing, and analysis

Using a 3 T Trio Tim scanner (Siemens) equipped with a 32-channel head coil, all MR images [T1- (T1w) and T2-weighted (T2w), resting-state functional MRI (fMRI) and diffusion-weighted (dMRI)] were collected from November 11, 2016, to March 3, 2018, at Tamagawa University Brain Science Institute. The parameters used in resting-state fMRI and dMRI acquisition are as described previously (Ishihara et al., 2021). The resting-state fMRI data and diffusion-weighted images were acquired twice with a reversed phase-encoding direction (anterior–posterior and posterior–anterior).

In the preprocessing and analysis of MRI data, the Functional Magnetic Resonance Imaging of the Brain (FMRIB) Software Library (FSL; version 5.0.9), FMRIB's ICA-based Xnoiseifier (FIX; version 1.062), FreeSurfer (version 5.3.0-HCP), Human Connectome Project (HCP) pipeline (version 3.22.0), and ConnectomeWorkbench (version 1.2.3) were used. The HCP structural preprocessing pipelines (Glasser et al., 2013), which include PreFreeSurfer, FreeSurfer, and PostFreeSurfer components, were used to analyze cortical structure. The PreFreeSurfer pipeline was utilized to (1) align and average repeated T1w and T2w scans of good or excellent quality (when available); (2) create an unbiased “native” volume space for each participant that is rigidly aligned to the Montreal Neurological Institute (MNI) template by removing gradient nonlinearity and readout distortion [static field (*b*0) distortion in three-dimensional images]; (3) perform cross-modal alignment between the T1w and T2w images by the use of FreeSurfer's boundary-based registration (BBR) method (Greve and Fischl, 2009); (4) perform bias field correction with the square root (T1w × T2w); and (5) perform nonlinear volume-based registration to the MNI template using FSL's FNIRT algorithm.

A customized version of FreeSurfer version 5.3 recon-all was used to generate white and pial cortical surfaces (using both T1w and T2w volumes at 0.8 mm resolution), including subcortical segmentation, all conducted in the participants’ native volume space. PostFreeSurfer was used to convert the FreeSurfer data into standard NIFTI, GIFTI, and CIFTI file formats and bring the data into the MNI space. To perform an initial, gentle, nonrigid surface registration based on folding patterns (MSMSulc), the Multimodal Surface Matching (MSM) surface registration algorithm (Robinson et al., 2014) was used. This technique has replaced the FreeSurfer folding-based registration utilized in earlier studies (Glasser et al., 2013) because it yields slightly better initial alignment of functionally corresponding regions (e.g., task fMRI) while producing significantly less local distortion than the FreeSurfer algorithm (Robinson et al., 2014). This registration together with the FNIRT nonlinear registration was used to bring an initial version of the data into standard grayordinate space (32-k standard mesh for each hemisphere's cortical surface at 2 mm average vertex spacing and 2 mm isotropic MNI space voxels for the subcortical volume data). The FreeSurfer-generated measure of cortical thickness was corrected for folding-related bias by regressing out the FreeSurfer mean curvature measure from each participant's thickness data (Glasser and Van Essen, 2011) because the gyral crowns tend to be thicker than the sulcal fundi. The ratio of T1w/T2w images, normalized for residual transmit field inhomogeneity, was used to compute myelin maps (Glasser and Van Essen, 2011; Glasser et al., 2013, 2014; Robinson et al., 2014).

All resting-state fMRI data were analyzed using the HCP functional preprocessing pipelines including volumetric- (fMRIVolume) and surface-based (fMRISurface) components (Glasser et al., 2013; Smith et al., 2013). The fMRIVolume pipeline included the following steps: removing gradient nonlinearity and *b*0 inhomogeneity-related image distortions; motion correction; cross-modal alignment to the T1w image with BBR (Greve and Fischl, 2009); concatenation of all transforms, including the nonlinear volume registration to MNI space; and resampling the original time series into MNI space using a single spline interpolation. Several intensity normalization steps were used, including a crude fMRI bias field correction step based on the structural data from a separate imaging session (this bias field correction is changed to a better method in the following processing steps, as described below, and has been incorporated into the most recent version of the pipelines) and grand four-dimensional mean normalization to 10,000. Next, to map gray matter time series data into the 91,282-grayordinate standard space (2 mm average cortical vertex spacing and 2 mm subcortical voxels) with a 2 mm full-width at half-maximum smoothing kernel (constrained to the cortical surface and subcortical gray matter segmentation), the fMRISurface pipeline was utilized. For each resting-state fMRI run, these steps produce a “dense time series” CIFTI file. To replace the crude bias field correction map with a better map, it was also mapped into standard CIFTI space (by dividing it back and multiplying it by the new correction map). For resting-state fMRI runs, independent component analysis and FMRIB's ICA-based Xnoisiefier (ICA + FIX) pipeline (Beckmann et al., 2005; Smith et al., 2013; Griffanti et al., 2014; Salimi-Khorshidi et al., 2014) were applied to resting-state fMRI scans to eliminate spatially specific temporally structured artifacts. The ICA + FIX pipeline included the following steps: (1) high-pass temporal filtering to eliminate linear trends in the data with a sigma of 1,000 s (run length, 864 s); (2) MELODIC ICA, for creating component spatial maps and time series with autodimensionality selection of up to 250 components; (3) the FIX-trained ICA component classifier, to categorize these components into signal and noise; and (4) out-regression of the data and all ICA components of the 24 motion parameters (which were also temporal high-pass–filtered with a sigma of 1,000 s). All ICA component time series were utilized to calculate regression coefficients, and the noise component time series were weighted by the coefficients and subtracted from the data (a “nonaggressive” regression approach). The volumetric time series data were processed through the ICA + FIX algorithms before the grayordinates time series data underwent the high-pass filter and nuisance regression stages. After the original, uncleaned, native mesh data had been resampled into the standard grayordinates space in accordance with the areal feature-based MSM surface registration, the ICA + FIX cleanup was again applied to the resting-state fMRI–dense time series data. Early analyses included regression of the mean gray signal (“global signal”); however, this method was abandoned because it shifted some resting-state fMRI functional connectivity gradient locations, thereby reducing cross-modal alignment. No further spatial smoothing or temporal low-pass filtering was performed because these types of “lossy” preprocessing steps would decrease the accuracy of the parcellations and proved unnecessary for the purposes of the current study. Using GraphVar (Kruschwitz et al., 2015), we subsequently developed participant-specific connection matrices from the time series signals of 360 brain regions. We employed the 360 areas that were defined by HCP-style parcellation (Glasser et al., 2016a,b) as nodes to build the brain network. Using Pearson's correlation coefficients, the edges of the brain network were defined as the functional connectivity of all pairs among the 360 regions.

HCP pipelines were also used to preprocess diffusion MRI data. Briefly, corrections for gradient, *B*0, eddy current distortions, and cross-modal registration were performed (Glasser et al., 2013; Sotiropoulos et al., 2013). The intensity was normalized by the mean of volumes with *b* = 0 s/mm^2^ (*b*0 volumes), and two opposing phase-encoded images and FSL's Topup (Andersson et al., 2003) were used to correct *B*0 inhomogeneity distortion. Before the recent recomputation of HCP, which included outlier detection (Andersson et al., 2012), FSL's Eddy version 5.0.9 was used to correct the eddy current-induced field inhomogeneities and head motion for each image volume. The data were then adjusted for gradient nonlinearity. The *b*0 volume and BBR cost function in FSL and FreeSurfer's BBRegister were used to register the diffusion data to the structural T1w anterior commissure–posterior commissure space and the white matter surface, respectively. Based on the rotational information of the *b*0 to the T1w transformation matrix, the diffusion gradient vectors were rotated.

To calculate the neurite orientation dispersion and density imaging (NODDI) coefficients, the AMICO toolbox was used. We modified the AMICO toolbox and changed its intrinsic free diffusivity parameter to 1.1 × 10^−3^ mm^2^/s for our analyses of gray matter structures because it is essential to optimize the NODDI model when analyzing gray matter structures since different types of the brain tissue may vary considerably regarding their intrinsic free diffusivity. Subsequently, we calculated the neurite density index (amount of stick-like or cylindrically symmetric diffusion produced when water molecules are constrained by neurite membranes) and orientation dispersion index (a tortuosity measure, coupling the intra- and extraneurite space, resulting in alignment or dispersion of axons and dendrites in the gray matter). Using NoddiSurfaceMapping (Fukutomi et al., 2018), the NODDI parameters were mapped onto the cortical surface. Using FSL's bedpostX and probtrackX methods (Behrens et al., 2007; Jbabdi et al., 2012), probabilistic tractography was performed to obtain a tractography matrix from the preprocessed diffusion MRI data. To obtain a tractography matrix of the number of streamlines originating from each region of interest (ROI) and reaching the rest of the cerebral cortex, we seeded 1,000 streamlines from each of the ROIs. By dividing each row by the waytotal file, the unnormalized values in these matrices were normalized.

We calculated the nodal graph measures using resting-state functional connectivity and tract-based structural connectivity matrix. Thresholding of the connectivity matrix was performed. To find the optimal threshold value, we performed a graph theory-based analysis (Khazaee et al., 2016) seeking a threshold value that maximized the global cost efficiency. We used segregation (clustering coefficient and local efficiency), integration (nodal path length), and centrality (degree and betweenness centrality) measures—the most commonly used concepts for describing the topological property of a node in a network. These nodal graph measures were calculated using GraphVar (Kruschwitz et al., 2015).

### Quantification and statistical analysis

All statistical analyses were conducted using RStudio (version 1.1.463). All variables were regressed from age and sex before analyses. Brain imaging variables were additionally regressed from handedness, intracranial volume, and transmitter reference amplitude. Multiple sparse canonical correlation analysis (MSCCA) was performed using the MultiCCA.permute and MultiCC functions in the PMA package to test the association of prosocial behavior with imaging data. Optimal weights and penalties were identified using the MultiCCA.permute function with 1,000 permutations. Before performing MSCCA, principal component analysis (PCA) was performed for dimensionality reduction in all sets of variables (i.e., economic games, cortical and subcortical structure, resting-state functional connectivity, and tractography-based structural connectivity) using the prcomp function. A dimensionality reduction step was performed to prevent an overdetermined, rank-deficient solution and eliminate the possibility of overfitting. The data obtained from economic games were reduced to 30 PCAs (variance explained, 89%), and all sets of MRI variables were reduced to 60 PCAs (variance explained, 64–72%). The permutation test confirmed whether the sum of canonical correlations was statistically significant relative to the null distribution. To produce the null distribution, we randomly shuffled the PCAs 1,000 times before performing MSCCA. The *p* value was calculated as the proportion of cases that showed a higher canonical correlation than the observed correlation, which was divided by 1,000. To confirm the robustness of MSCCA when varying the number of principal components, we repeated the whole statistical testing for a range of PCs (i.e., 70 and 80) for MRI variables. The results of these analyses are shown in Extended Data Figures 1-2 and 1-3.

To ensure the robustness of the results against additional potential confounders, we repeated the MSCCA while controlling for a comprehensive set of variables known to correlate with both prosocial behavior and brain structure (De Neys et al., 2011; Matsumoto et al., 2016; Piff and Robinson, 2017; van den Akker et al., 2020; Moya and Alcañiz-Colomer, 2023). The aforementioned factors included the education level (determined by completion of an undergraduate degree) and income measured via a seven-point scale where 1 is none, 2 is <1.5 million yen, 3 is <3 million yen, 4 is <5 million yen, 5 is <7 million yen, 6 is <10 million yen, and 7 is 10 million yen or more. The second variable was IQ, measured using the Kyoto University NX-15 (Osaka and Umemoto, 1984). The third variable was cognitive task performance, measured using the go/no-go task false alarms (Ishihara et al., 2021).

### Resource availability

#### Lead contact

Further information and requests for resources should be directed to and will be fulfilled by the lead contact, Haruto Takagishi (takagishi@lab.tamagawa.ac.jp).

#### Materials availability statement

This study did not generate new unique reagents.

#### Data and code availability

This paper analyzes existing, publicly available data. These accession numbers for the datasets are listed in the key resources table. This paper does not report the original code. All data are available from the Dryad Digital Repository (Ishihara et al., 2025): https://doi.org/10.5061/dryad.gxd2547tb.

## Results

In total, 217 participants aged 20–60 years participated in 15 major economic games and underwent MRI between 2012 and 2018. Participants played economic games in a completely anonymous environment and received rewards based on their actual behavior. Structural (T1w and T2w), resting-state functional, and diffusion MRI data were collected and preprocessed using the HCP pipeline tools (Glasser et al., 2016a) to obtain data for all 360 cortical and 41 subcortical brain regions, including data on cortical thickness, T1w/T2w myelin maps, neurite orientation dispersion, neurite density, functional and structural connectivity based on graph theory indices (centrality, segregation, integration, and interhemispheric connection), and subcortical volume. A comprehensive analysis of the association between 108 behavioral data points from 15 economic games and data from 5,441 MR images was conducted using sparse multiple canonical correlation analysis (SMCCA) after dimension reduction using PCA.

**Figure 1.**
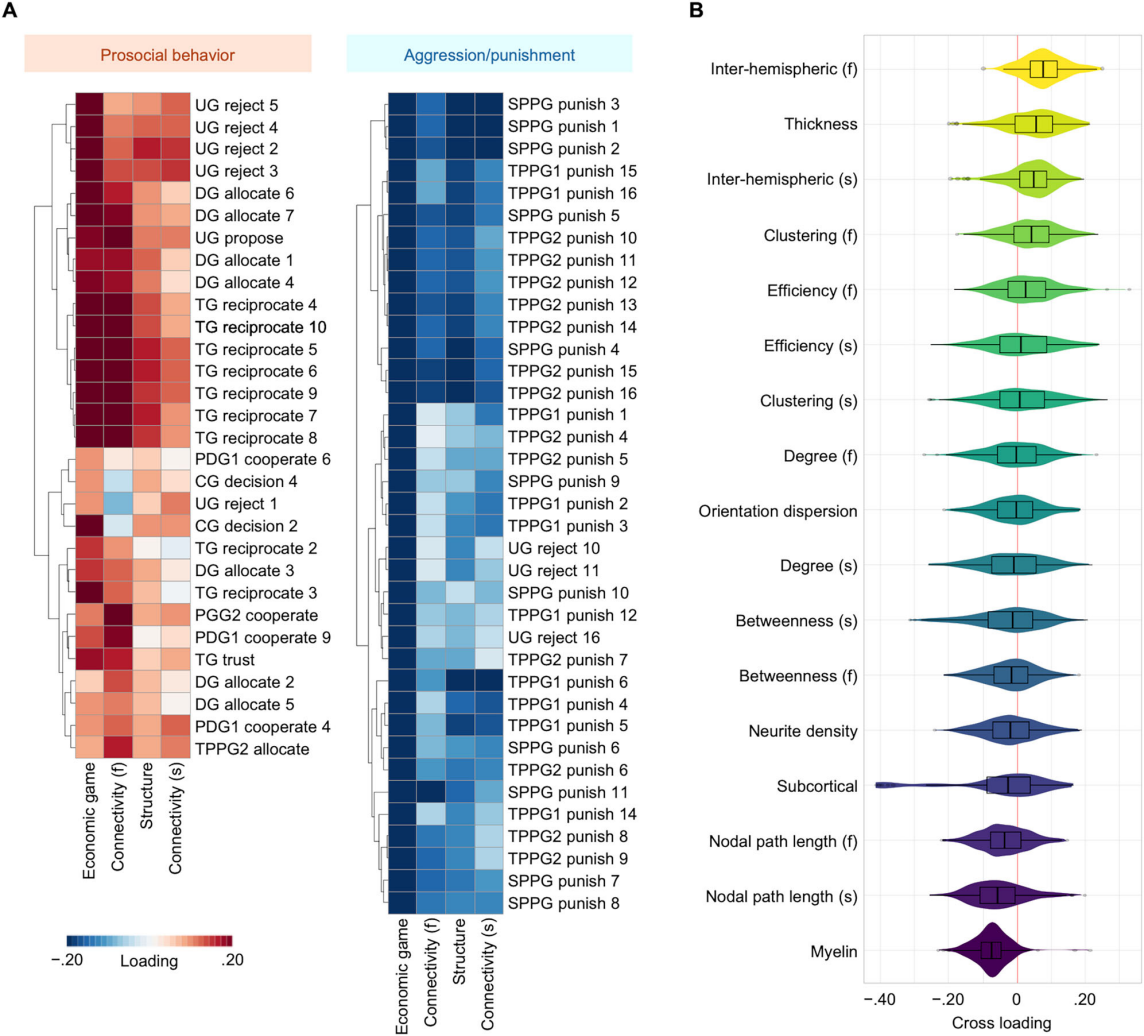
Results of MSCCA. ***A***, Canonical cross-loadings for economic games. Canonical loadings for economic games are presented only in clusters with consistent positive loadings (left panel) and negative loadings (right panel), which reflect prosocial behavior and aggression/punishment, respectively. The full results are presented in Extended Data Figure 1-1. ***B***, Mean canonical cross-loadings for brain imaging data. Canonical cross-loadings of the MRI dataset (5,441 images) are divided into each MRI metric and presented as violin and box plots. (f) denotes measures of resting-state functional connectivity; (s) denotes measures of tractography-based structural connectivity. Descriptions of the variables measured in each economic game are presented in Extended Data Table 1-2. The results after controlling for potential confounding factors are shown in Extended Data Figures 1-2 and 1-3. UG, ultimatum game; DG, dictator game; TG, trust game; PDG, prisoner's dilemma game; CG, chicken game; PGG, public goods game; TPPG, third-party punishment game; SPPG, second-party punishment game; MRI, magnetic resonance imaging.

Economic games’ variates correlated with variates for cortical and subcortical structure (*ρ* = 0.47) and functional (*ρ* = 0.35) and structural (*ρ* = 0.40) connectivity (nonparametric permutation test, *p* = 0.004). Canonical cross-loading for economic games showed that prosocial behavior and aggression/punishment had positive and negative loadings, respectively, with brain imaging variates (Fig. 1*A*; Extended Data Table 2-1). Canonical cross-loadings to economic games from brain imaging data consistently showed positive loadings of interhemispheric connections [interhemispheric functional connectivity: mean, 0.08; 95% confidence interval (CI), 0.07–0.09; 90% of regions showed positive loadings; interhemispheric structural connectivity: mean, 0.04; 95% CI, 0.03–0.05; 78% of regions showed positive loadings; corpus callosum volume: mean, 0.08; 95% CI, 0.06–0.10; all regions showed positive loadings; Fig. 1*B*; Extended Data Table 2-1]. Cortical T1w/T2w myelin maps had strong negative cross-loadings (mean, −0.08; 95% CI, −0.08 to −0.07; 96% of regions showed negative loadings; Fig. 1*B*; Extended Data Table 2-1). The positive cross-loadings were detected for cortical thickness (mean, 0.05; 95% CI, 0.04–0.06; 72% of regions showed positive loadings) and certain subcortical regions’ volume (nucleus accumbens, amygdala, hippocampus, and pallidum), while the ventricle showed negative loadings (mean, −0.30; 95% CI, −0.36 to −0.24; all regions showed negative loadings). The segregation measures of functional connectivity showed positive loadings (clustering coefficient: mean, 0.04; 95% CI, 0.03–0.05; local efficiency: mean, 0.03; 95% CI, 0.02–0.04; 72 and 63% of regions showed positive loadings, respectively), while an integration measure (nodal path length) showed negative cross-loadings (functional connectivity: mean, −0.04; 95% CI, −0.04 to −0.03; structural connectivity: mean, −0.05; 95% CI, −0.06 to −0.04; 71 and 77% of regions showed negative loadings, respectively; Fig. 1*B*; Extended Data Table 2-1). Other measures did not show a consistent association with economic game variety.

10.1523/ENEURO.0304-24.2025.f1-1 Figure 1-1 Complete list of canonical cross-loadings for prosocial behaviors. Descriptions of the variables measured in each economic game are presented in Table 1-2. Download Figure 1-1, TIF file.

**Figure 2.**
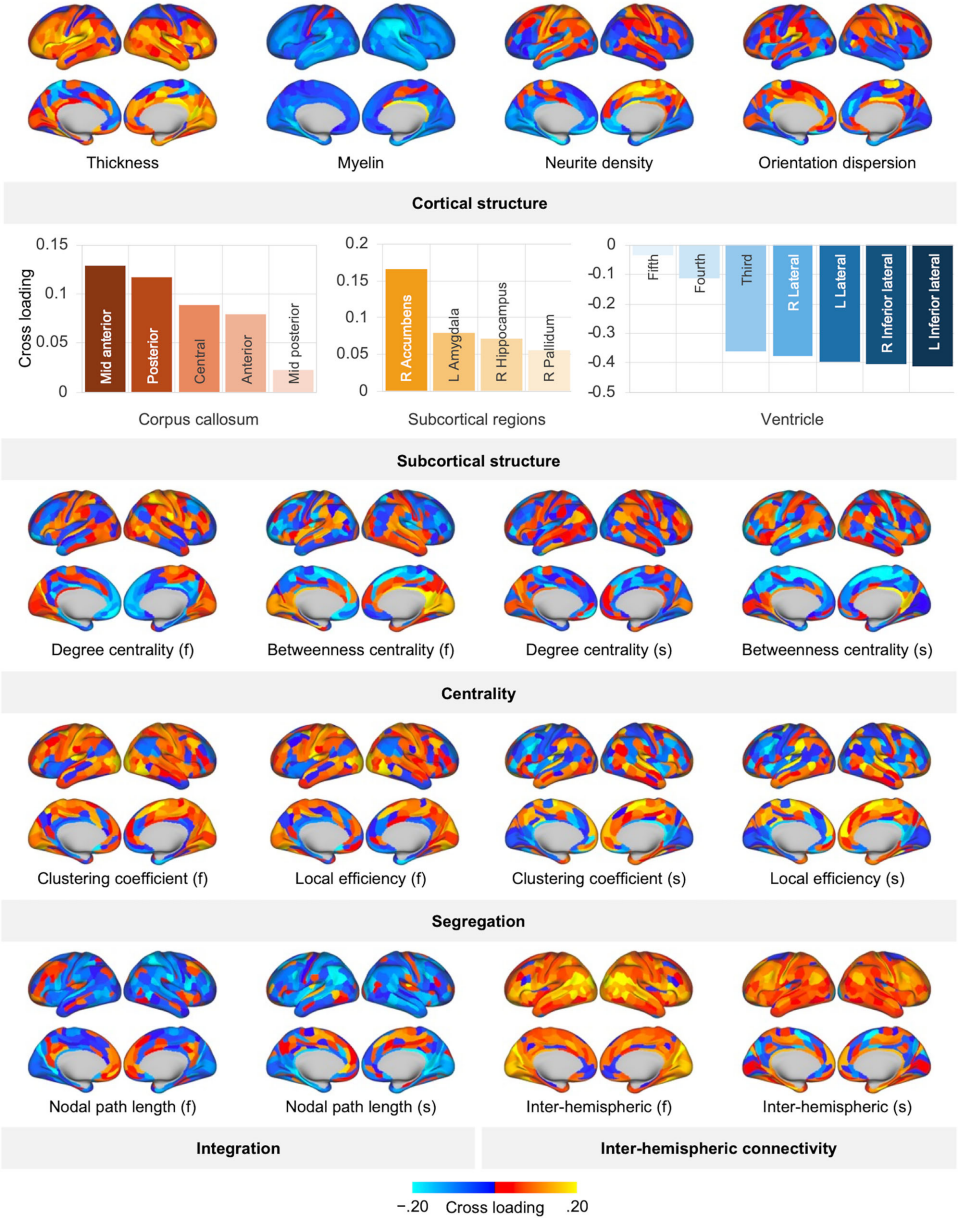
Canonical cross-loadings for brain imaging data in each region. Only the canonical cross-loading most strongly associated with the economic games variate is presented for subcortical volume (for more quantitative results, please refer to Extended Data Table 2-1). (f) denotes measures of resting-state functional connectivity; (s) denotes measures of tractography-based structural connectivity.

10.1523/ENEURO.0304-24.2025.f1-2 Figure 1-2 Results of canonical cross-loadings for prosocial behavior and brain imaging data with variation in the number of principal components and adjustments for potential confounders. (A) Canonical cross-loadings for prosocial behavior, and (B) mean canonical cross-loadings for brain imaging data. Descriptions of the variables measured in each economic game are presented in Table 1-2. Download Figure 1-2, TIF file.

**Figure 3.**
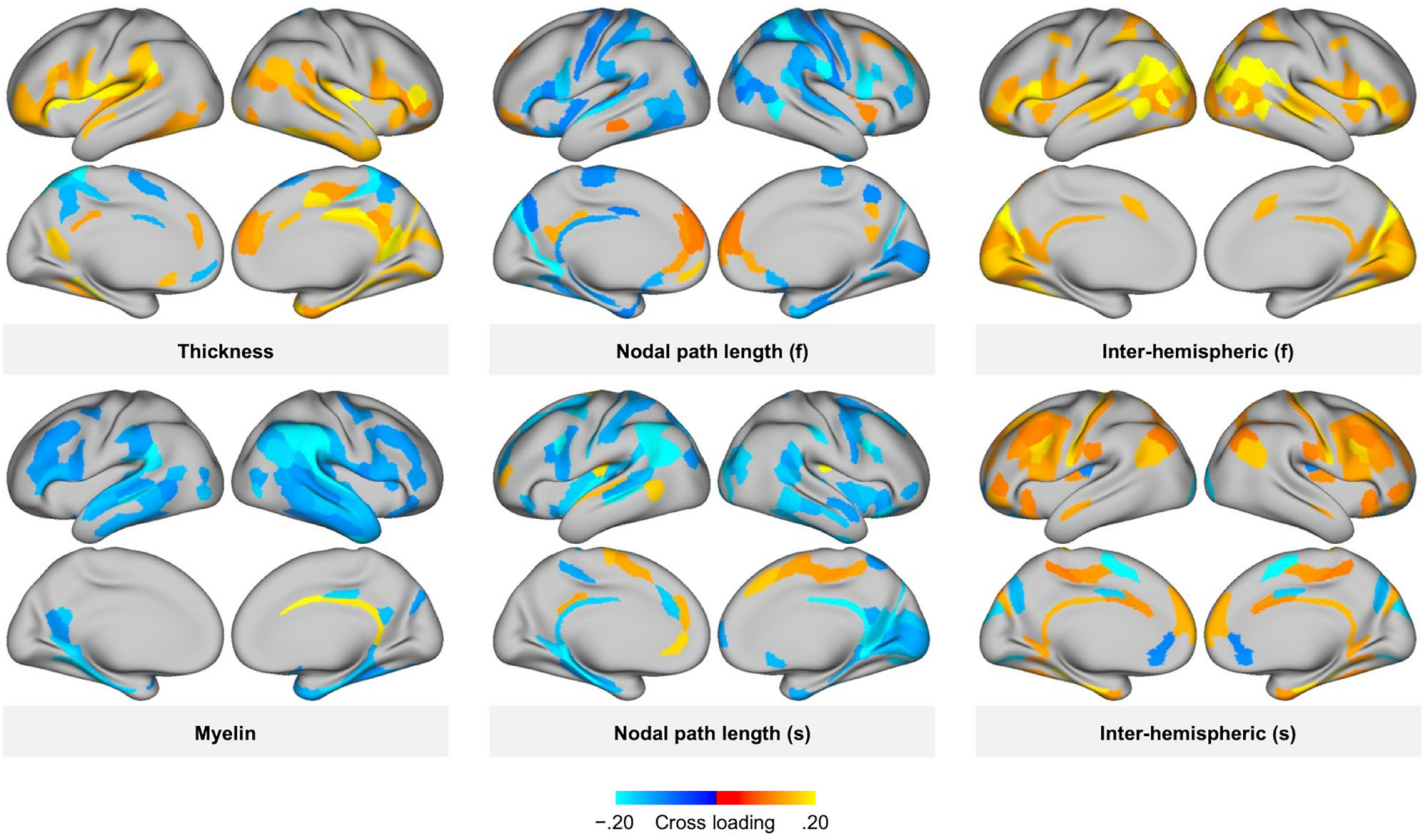
A brain regional pattern of the strong covariation of brain imaging data with economic games. This figure maps brain regions that show only the top 30% canonical correlation coefficients to confirm the brain regions strongly associated with prosociality. (f) denotes measures of resting-state functional connectivity; (s) denotes measures of tractography-based structural connectivity. Relationships between other brain indicators and economic games are shown in Extended Data Figure 3-1.

10.1523/ENEURO.0304-24.2025.f1-3 Figure 1-3 Correlation among canonical cross-loadings for brain imaging data in each index with variation in the number of principal components and adjustments for potential confounders. Download Figure 1-3, TIF file.

10.1523/ENEURO.0304-24.2025.t1-1 Table 1-1 List of papers published in this research project. Download Table 1-1, XLS file.

10.1523/ENEURO.0304-24.2025.t1-2 Table 1-2 Description of variables used in the analysis. Download Table 1-2, XLS file.

The inter-region variations of canonical cross-loading to economic games are presented in Figure 2. As cortical thickness must be considered for the interpretation of individual differences in cortical T1w/T2w myelin map (Glasser et al., 2022), a correlation analysis was performed. The cross-loading of T1w/T2w myelin maps inversely correlated with that of cortical thickness (Pearson's *r*, −0.23; 95% CI −0.32 to −0.13). When imaging data were summarized according to the brain regions most strongly contributing to the prosocial behavior (top 30%), MRI data strongly covaried with economic games (i.e., cortical thickness, T1w/T2w myelin maps, nodal path length, and interhemispheric connectivity), and a pattern emerged (Fig. 3) that included the temporal, parietal, insula, and inferior frontal regions, which are well known as social brain networks. However, no such specific patterns were seen for the remaining MRI indications (Extended Data Fig. 3-1). The pre-SMCCA dimension reduction using PCA was run using a variable number of PCAs and adjustments for additional potential confounders (education level, income, IQ, and cognitive task performance), with almost no change in the results (Extended Data Figs. 1-2, 1-3).

10.1523/ENEURO.0304-24.2025.t2-1 Table 2-1 Complete list of canonical loadings. Download Table 2-1, XLS file.

10.1523/ENEURO.0304-24.2025.f3-1 Figure 3-1 A brain regional pattern of the strong covariation of brain imaging data with economic games. This figure maps brain regions that show only the top 30% canonical correlation coefficients to confirm the brain regions strongly associated with prosociality. (f) denotes measures of resting-state functional connectivity; (s) denotes measures of tractography-based structural connectivity. Download Figure 3-1, TIF file.

## Discussion

The comprehensive data-driven analysis using SMCCA identified brain functions and structures that support prosocial behavior. The higher the degree of prosocial behavior, the greater the functional connectivity between cerebral hemispheres. Regarding brain function during resting state, we found that the degree of prosocial behavior was higher in individuals with a better functional balance in the same brain region between the cerebral hemispheres. Additionally, individuals with stronger structural connectivity between hemispheres and larger corpus callosum volumes exhibited higher degrees of prosocial behavior. These findings are consistent with those of previous research indicating that patients with autism spectrum disorders, primarily characterized by social deficits, have weaker resting-state functional- and tractography-based structural connections between hemispheres (Anderson et al., 2011; Lo et al., 2011) and smaller corpus callosum volumes (Travers et al., 2012). Moreover, the negative association between ventricular volume and degree of prosocial behavior in the present study is consistent with previous findings that patients with autism spectrum disorders have larger ventricular volume (Turner et al., 2016). The corpus callosum is a bundle of white matter fibers connecting the left and right hemispheres. Compared with chimpanzees, humans have a larger volume of the rostral body of the corpus callosum, involved in behavioral control (Sakai et al., 2017), and humans exhibit higher degrees of prosocial behavior than chimpanzees (Warneken and Tomasello, 2009; Silk and House, 2011). Altogether, this evidence suggests that the evolution of the interhemispheric connections supported by structural connectivity, and the large volume of the corpus callosum, might explain why humans are more prosocial than other animals and allude to the critical factors that separate humans from other animals.

Following the interhemispheric functional and structural connectivity, decreased T1w/T2w myelin maps were also associated with a higher degree of prosocial behavior. Myelin insulates the axons of neurons and is involved in the efficiency of neurotransmission (Hartline, 2008; Stadelmann et al., 2019). Generally, lower T1w/T2w myelin maps indicate demyelination of the axon; however, the association of T1w/T2w myelin maps with prosocial behavior should be interpreted along with the results of cortical thickness. A higher degree of prosocial behavior was found to be associated with a thicker cortex. Decreased myelination along with increased cortical thickness may be due to increased nonmyelinated plasticity supporting cellular constituents, including dendrites, spines, synapses, and glia, and not necessarily due to an absolute decrease in the degree of myelination (Glasser et al., 2022). Therefore, one or more of these nonmyelinated constituents play(s) an important role in promoting human prosocial behavior.

The covariation of interhemispheric connectivity, T1w/T2w myelin maps, and cortical thickness with prosocial behavior was found in the whole brain; however, it was more pronounced in the social brain network (Blakemore, 2008), including the temporal lobe, TPJ, insula, and inferior frontal gyrus. These findings support those of previous studies showing the prominent role of social brain networks in human prosocial behavior (Morishima et al., 2012; Kameda et al., 2016). Previous studies on cortical thickness have found that people with the thicker dorsolateral prefrontal cortex (DLPFC) have a lower allocation in the DG (Yamagishi et al., 2016), suggesting that the DLPFC inhibits prosocial impulses. As the current study used some of the same data as those used previously (Yamagishi et al., 2016), at first glance, the present results may appear to contradict those of the aforementioned study. However, the prosocial component was extracted from various economic games, not only the DG, and the relationship between various MRI indices in the whole brain was examined. In addition, unlike the current study, previous studies focused on the DLPFC and did not report any association with cortical thickness in the social brain regions, including the TPJ, which was found to be relevant in the present study. In this study, the association between prosocial behavior and DLPFC was relatively weak and not necessarily contradictory because it was more closely related to the social brain regions, such as the TPJ and insula, as described above.

Several graph theory indicators also showed associations with prosocial behavior. First, a higher clustering coefficient and local efficiency, as well as a shorter nodal path length, were associated with a higher degree of prosocial behavior. These results indicate that the higher the modular segregation and quicker the inter-regional communications, the higher the prosocial behavior, i.e., the more efficient it is. The association between integration and prosocial behavior was prominent in the social brain regions, while no such pattern was found for segregation, suggesting that human prosociality is supported by a social brain network that allows rapid communication with other regions. Although the relationship between TPJ and prosocial behavior has been previously reported (Morishima et al., 2012; Kameda et al., 2016), the present study significantly advances our understanding by demonstrating that heightened prosocial behavior is achieved by rapid communication of social brain-related regions, including the TPJ.

A data-driven analytical approach was used in the current study to successfully extract brain functions and structures associated with prosocial behavior. Notably however, the term “prosocial behavior” encompasses a range of concepts, including altruism, fairness, and reciprocity. As a result, it is uncertain precisely which aspects of prosocial behavior the results of this study are indicative of. The extracted components from the various economic games demonstrated positive loadings for reciprocity in the TG and altruism in the DG, as well as negative loadings for third-party punishment and second-party punishment. The question arises as to what conceptual elements are reflected in these components. For example, fairness is thought to be a concept common to such decisions, although it could also be thought to reflect decision-making in situations where one's own reward is not affected by the decisions of others.

CCA is an effective method for extracting relationships between a large number of variables, although interpreting results obtained from it can be challenging. The approach does not allow for definitive conclusions to be drawn regarding the extent to which results reflect prosocial behavior nor does it permit an accurate determination of the specific aspects of prosocial behavior that are reflected. In the next stage of the study, the theoretical classification of the decision-making process observed in the aforementioned economic game will be useful. Following this, it is hoped that a CCA will be conducted for each classification, with the aim of identifying brain regions and indicators common to each classification, as well as brain regions and indicators unique to each classification.

This study used multimodal brain measures and many major economic game measures to identify brain functions and structural bases underlying prosocial behavior in over 200 adults. This approach, called population neuroscience (Okada et al., 2019), uses a large amount of MRI data of many participants, and we believe that it is effective for obtaining more robust results. However, dealing with large amounts of data involving multisets of multiple variables can present a disadvantage as the interpretation becomes complex when using repeated analyses, such as one-to-one or many-to-one relationships, including Pearson's correlation coefficient and multiple regression. Furthermore, repeated analysis should be avoided due to the risks of Type 1 and Type 2 errors. To address these analytical issues, the SMCCA approach is effective as it allows the identification of patterns that describe many-to-many relationships. Based on the present results obtained using these approaches, we conclude that the neural basis of human prosocial behavior involves strengthened interhemispheric connections, a larger cortex due to increased nonmyelinated tissues, and functional segregation and integration in the social brain network. The study provides evidence that the human brain is uniquely adapted to support prosocial behavior, which may have contributed to the evolution of human sociality.

## Synthesis

Reviewing Editor: Alexander Soutschek, Ludwig-Maximilians-Universitat Munchen Decisions are customarily a result of the Reviewing Editor and the peer reviewers coming together and discussing their recommendations until a consensus is reached. When revisions are invited, a fact-based synthesis statement explaining their decision and outlining what is needed to prepare a revision will be listed below. The following reviewer(s) agreed to reveal their identity: Arkady Konovalov, Pyungwon Kang. Note: If this manuscript was transferred from JNeurosci and a decision was made to accept the manuscript without peer review, a brief statement to this effect will instead be what is listed below.

Reviewer #1

The paper presents the results of a secondary data analysis from a larger study (16 papers published to date) where several hundred subjects underwent MRI scans and played a series of economic games that tested prosocial behavior (the Dictator game, the Prisoner's Dilemma, the Ultimatum game, etc.). The paper presents the analysis of the link between the structure of the brain and prosocial behavior in 217 subjects.

Overall, the report is short and clear, the methods look robust and the results contribute to the previously published set of findings. The methods are clearly described, the presentation of results is clear, and the discussion contributes to the literature on the topic.

I have only two major questions/comments that might require some re-analysis of the data but - I hope - would make the findings more robust and more interesting to the general audience.

(1) First, the methods mentioned that age and sex were regressed out, but prosocial behavior can also be driven (or associated with) other demographic variables such as income, wealth, and cognitive ability, just to name a few. As I understand, a large set of data was collected for each subject, so I believe it is important to control for as many confounds as the data allows besides just age and sex. I hope additional controls can be included in a revision.

(2) Second, the findings are purely correlational, but given the large sample size and the depth of analysis it seems to be possible to use these data to predict an individual's prosocial behavior across games based on their structural characteristics. I believe including such an out-of-sample prediction exercise would make the paper's contribution stronger and more appealing to the general audience.

Reviewer #2

This paper introduces an interesting study addressing the neural markers associated with prosocial behavior, utilizing multimodal imaging data and incorporating a comprehensive array of economic games. This approach offers a broad and extensive dataset, making a significant contribution to understanding the neural basis of prosocial behavior. The study tackles a critical question in the field with a diverse methodological framework. However, there are several shortcomings that need to be addressed before the publication. These issues should be carefully addressed and resolved to enhance the impact of the findings.

1. Although prosociality is a crucial concept that has been studied extensively in Economics, it encompasses various behaviors such as altruism, reciprocity, fairness consideration, and trust, as mentioned by the authors in the introduction. The study includes multiple economic games to assess all these behaviors. However, I doubt that the PCA results related to the economic games can serve as a core variable representing all types of prosocial behaviors. The positive and negative loadings about economic decisions seem to distinguish between prosocial and punishment behaviors. Still, the loadings representing prosocial behavior appear to be driven by several components that assess similar concepts (such as reciprocity or fairness consideration) rather than providing an unbiased, abstract level of representation for prosocial behavior. When investigating neural markers for prosocial behavior, it would be ideal to examine more concrete concepts such as altruism and reciprocity separately or attempt to extract more comprehensive and representative variables for prosocial behavior. With the current results, it is difficult to interpret whether the findings reflect general prosocial behavior or are related to more specific concepts that drove the loadings for prosocial behavior. While addressing this issue fully could warrant another paper, I strongly recommend discussing this concern and other possible interpretations. Additionally, include a discussion about the conceptual connections between prosociality and its constituent behaviors.

2. On lines 69-71, the term "social brain" is ambiguous, and it is unclear what the authors intended to explain. This section should be elaborated, introducing more clearly what the "social brain" entails.

3. On line 104, the phrase "several participants simultaneously" needs to be replaced with an exact number. Provide the range of participants.

4. In economic games that involve the opponent's decision, it must be specified when the participants learned about the opponent's decision, especially if the game was repeated.
