# Supplementary figures and images for: Multimodal Imaging to Identify Brain Markers of Human Prosocial Behavior
Source: eNeuro. 2025 Feb 28;12(3):ENEURO.0304-24.2025. doi: 10.1523/ENEURO.0304-24.2025 (PMC11884870; doi:10.1523/ENEURO.0304-24.2025)

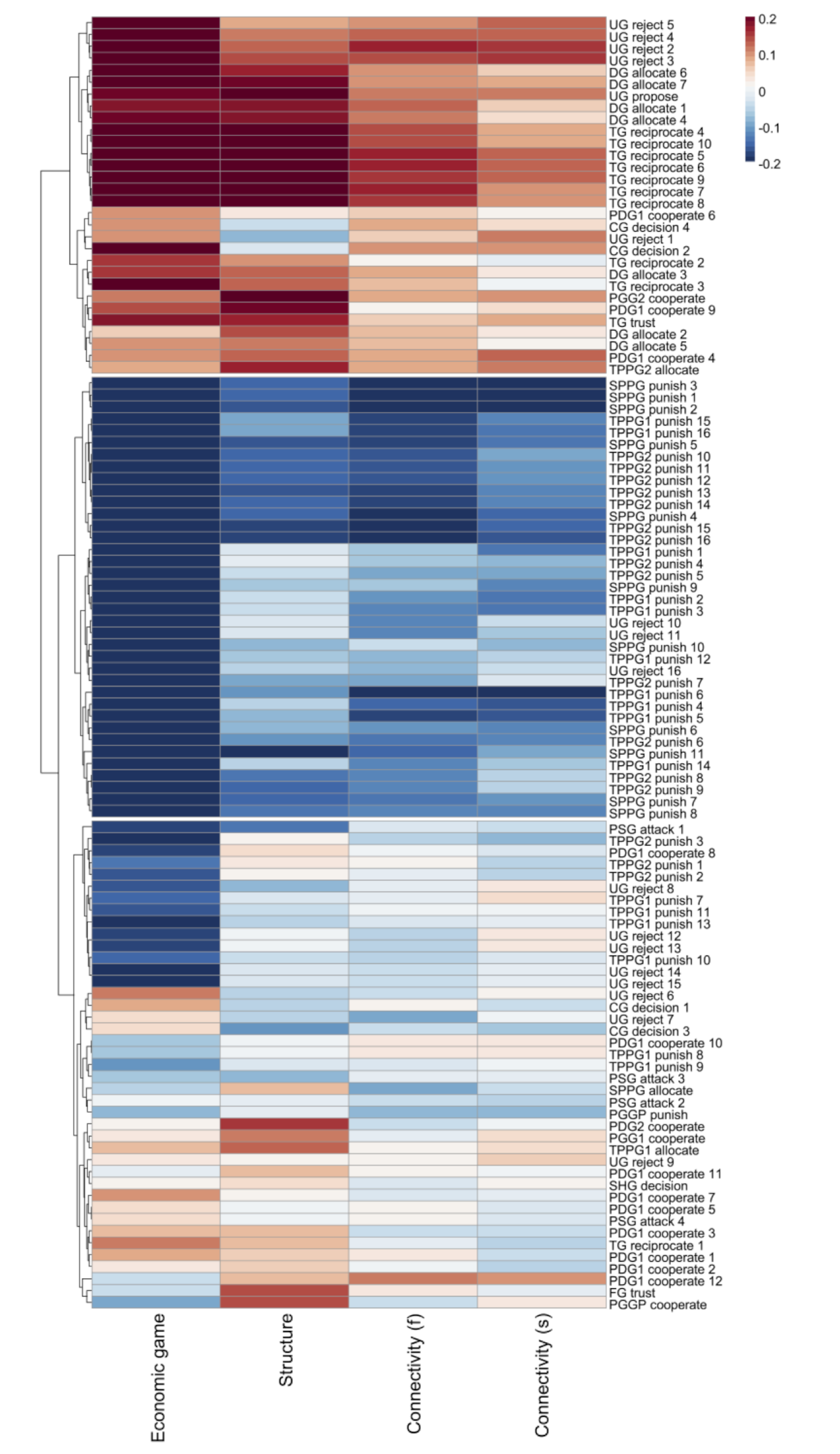

Supplement: Figure 1-1 — Complete list of canonical cross-loadings for prosocial behaviors. Descriptions of the variables measured in each economic game are presented in Table 1-2. Download Figure 1-1, TIF file. [file eneuro-12-ENEURO.0304-24.2025-s001.tif]

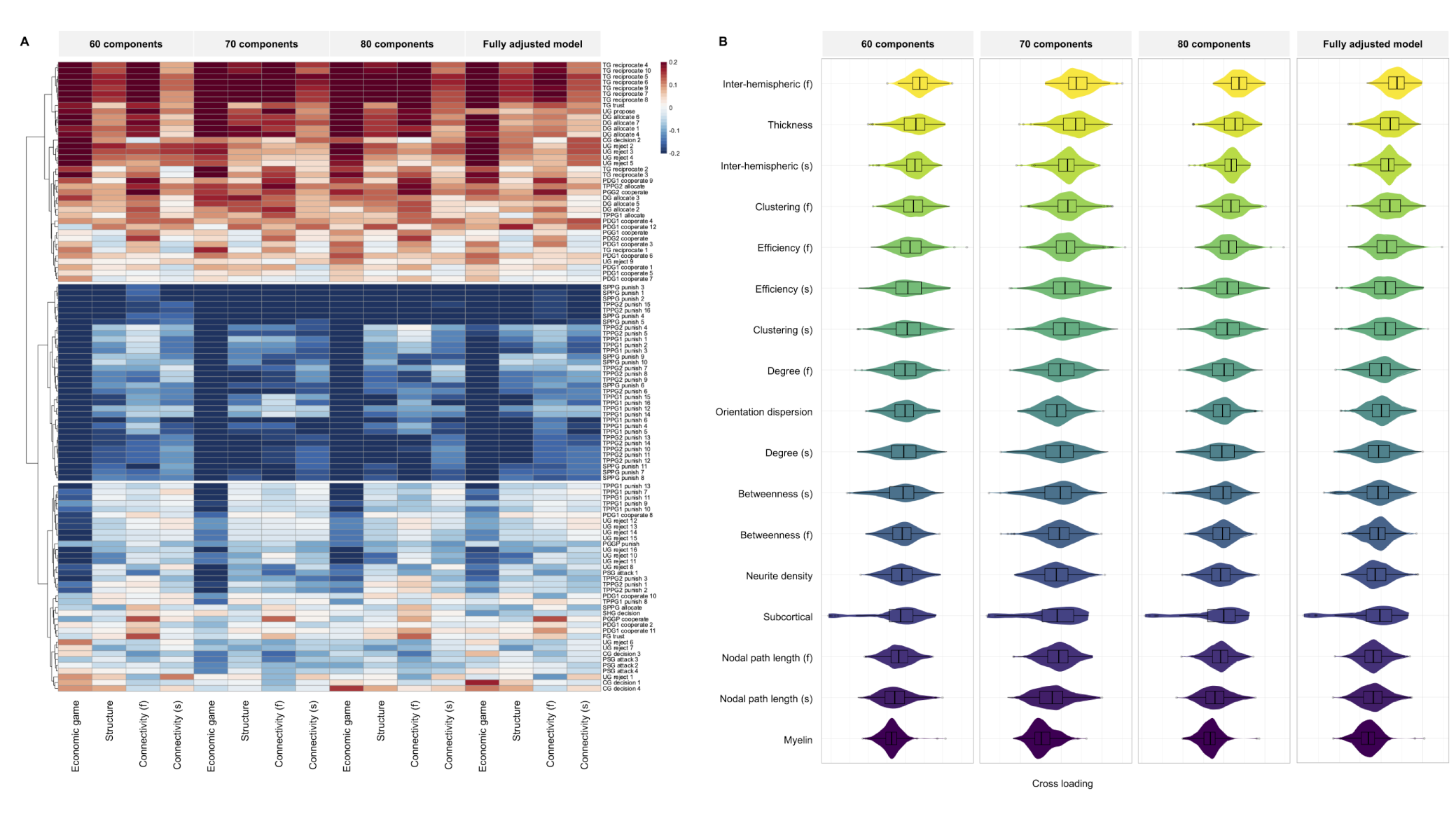

Supplement: Figure 1-2 — Results of canonical cross-loadings for prosocial behavior and brain imaging data with variation in the number of principal components and adjustments for potential confounders. (A) Canonical cross-loadings for prosocial behavior, and (B) mean canonical cross-loadings for brain imaging data. Descriptions of the variables measured in each economic game are presented in Table 1-2. Download Figure 1-2, TIF file. [file eneuro-12-ENEURO.0304-24.2025-s002.tif]

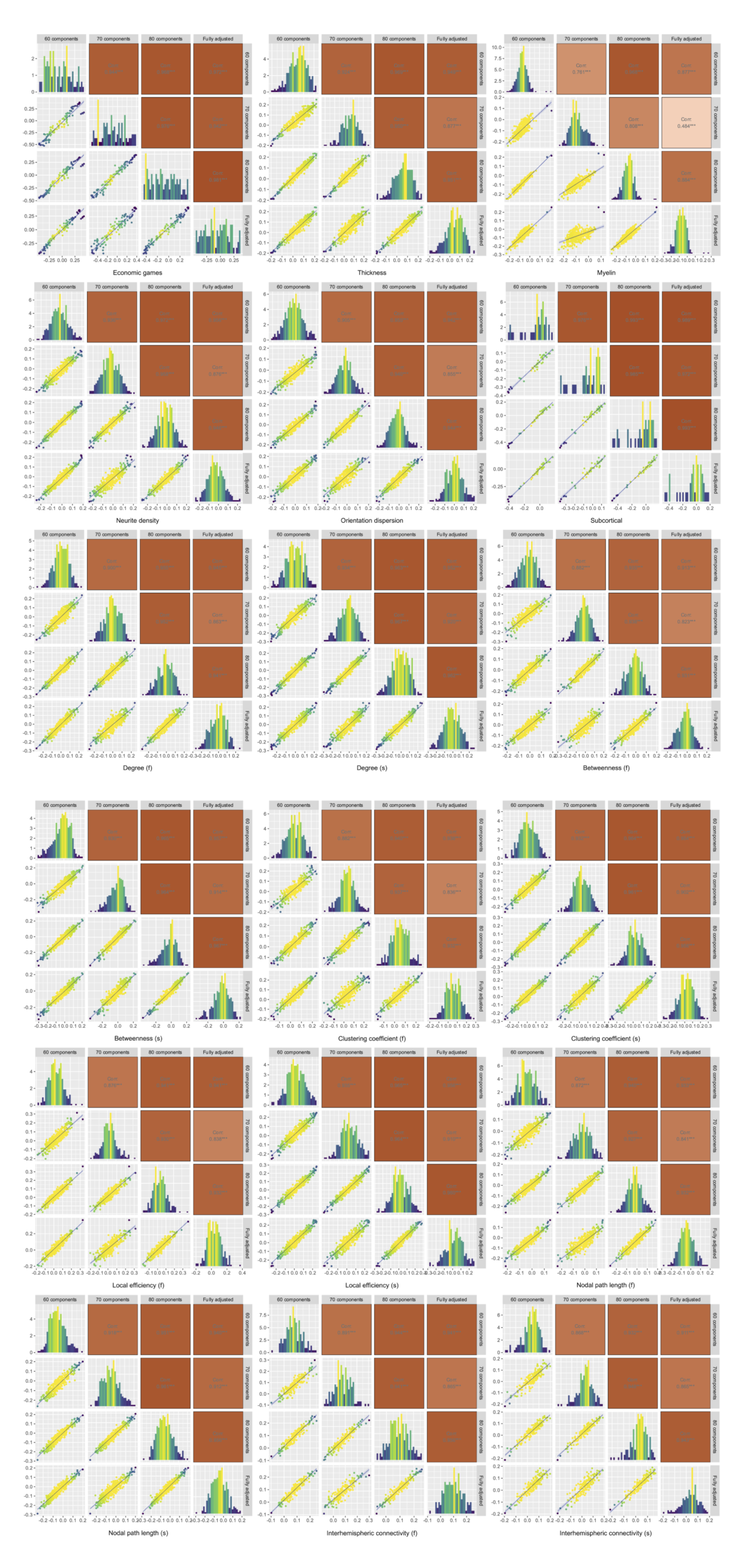

Supplement: Figure 1-3 — Correlation among canonical cross-loadings for brain imaging data in each index with variation in the number of principal components and adjustments for potential confounders. Download Figure 1-3, TIF file. [file eneuro-12-ENEURO.0304-24.2025-s003.tif]

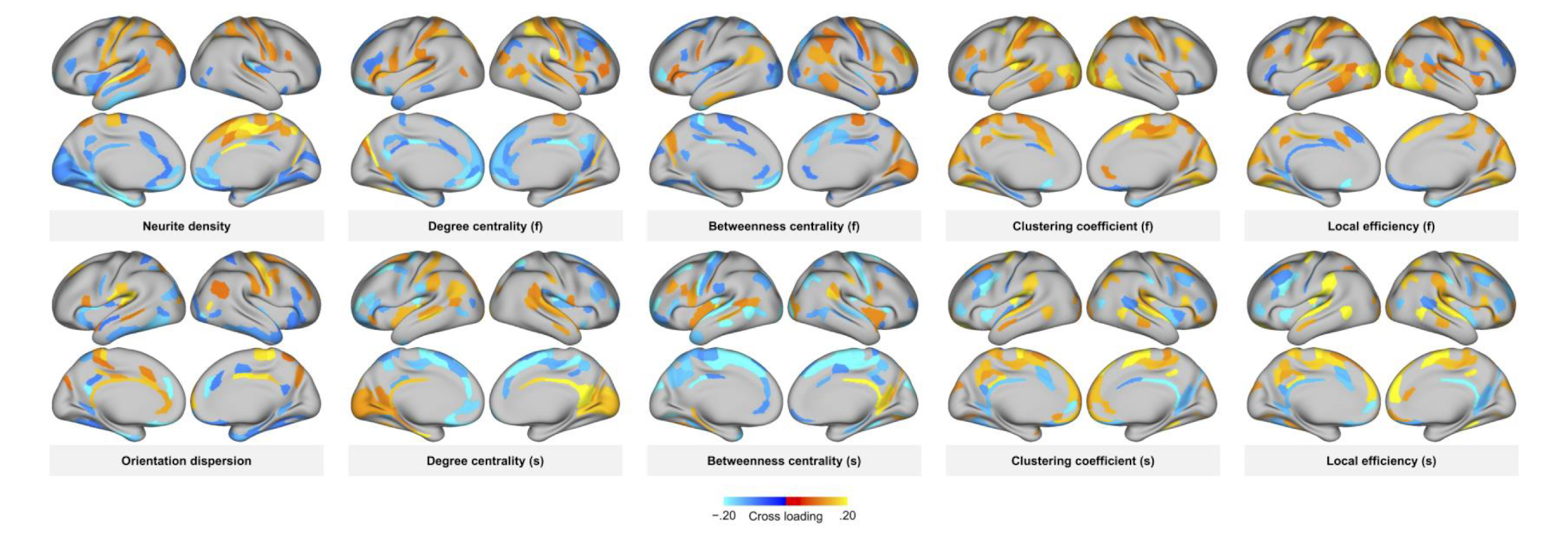

Supplement: Figure 3-1 — A brain regional pattern of the strong covariation of brain imaging data with economic games. This figure maps brain regions that show only the top 30% canonical correlation coefficients to confirm the brain regions strongly associated with prosociality. (f) denotes measures of resting-state functional connectivity; (s) denotes measures of tractography-based structural connectivity. Download Figure 3-1, TIF file. [file eneuro-12-ENEURO.0304-24.2025-s004.tif]
